# Mapping Physician Twitter Networks: Describing How They Work as a First Step in Understanding Connectivity, Information Flow, and Message Diffusion

**DOI:** 10.2196/jmir.3006

**Published:** 2014-04-14

**Authors:** Ranit Mishori, Lisa Oberoi Singh, Brendan Levy, Calvin Newport

**Affiliations:** ^1^Department of Family Medicine's Center for Health Communication, Media and Primary CareGeorgetown University School of MedicineWashington, DCUnited States; ^2^Department of Computer ScienceGeorgetown UniversityWashington, DCUnited States; ^3^Georgetown University/Providence Hospital Residency in Family MedicineColmar Manor, MDUnited States

**Keywords:** social networking, network analysis, information science, dissemination science, infodemiology, physician communication, physician networks, Twitter

## Abstract

**Background:**

Twitter is becoming an important tool in medicine, but there is little information on Twitter metrics. In order to recommend best practices for information dissemination and diffusion, it is important to first study and analyze the networks.

**Objective:**

This study describes the characteristics of four medical networks, analyzes their theoretical dissemination potential, their actual dissemination, and the propagation and distribution of tweets.

**Methods:**

Open Twitter data was used to characterize four networks: the American Medical Association (AMA), the American Academy of Family Physicians (AAFP), the American Academy of Pediatrics (AAP), and the American College of Physicians (ACP). Data were collected between July 2012 and September 2012. Visualization was used to understand the follower overlap between the groups. Actual flow of the tweets for each group was assessed. Tweets were examined using Topsy, a Twitter data aggregator.

**Results:**

The theoretical information dissemination potential for the groups is large. A collective community is emerging, where large percentages of individuals are following more than one of the groups. The overlap across groups is small, indicating a limited amount of community cohesion and cross-fertilization. The AMA followers’ network is not as active as the other networks. The AMA posted the largest number of tweets while the AAP posted the fewest. The number of retweets for each organization was low indicating dissemination that is far below its potential.

**Conclusions:**

To increase the dissemination potential, medical groups should develop a more cohesive community of shared followers. Tweet content must be engaging to provide a hook for retweeting and reaching potential audience. Next steps call for content analysis, assessment of the behavior and actions of the messengers and the recipients, and a larger-scale study that considers other medical groups using Twitter.

## Introduction

### Background

Social media, including Facebook and Twitter, is fast becoming an important tool in health care. In editorials, essays, and blogs, physicians have been urged to become active participants in social media as a form of engagement with the larger health community, patients, and peers [1], and as a way to “start an online dialogue” with policy makers and stakeholders [2]. Twitter, the microblogging medium, has been hailed as “an essential tool for every physician leader” [3], one that is “crucial to the development of medicine today” [4], and “just what the doctor ordered” [5].

This rapidly growing social network has approximately 500 million users worldwide, 140 million of them in the United States [6]. Some say it has “democratized” health information exchanges because is it a highly participatory medium where patients, physicians, health care organizations, and other stakeholders can interact on equal footing. Social media, including Twitter, has been described as one of the main tenets of what some scientists have called Medicine 2.0, or “next generation medicine” [7]. Still, despite the increasing interest and the huge potential for information diffusion and its analysis, it is not entirely clear who in the medical world is using Twitter, how much they are using it, and for what purpose [8].

In order to make inferences about group behavior and predictions about “best practices” for dissemination and diffusion of information through these networks, it is important to analyze the networks first. Social network analysis (SNA) is a well-established technique in sociology that can be adapted and used to systematically explore virtual communities, such as those that exist within the world of medicine. Applied graph theory is an overlapping area focused on using graphs to represent structures and networks, and theory developed about graphs to explain applications in a variety of fields, from computer science, to biology and chemistry, to mathematics and linguistics, to name a few. SNA and applied graph theory have been used to analyze structural patterns of social relationships, to explore influential information brokers, and to visualize the formal or informal personal networks within and between organizations.

Online networks have been studied before in relation to their topological structure, patterns of propagation of information, homophily (the tendency of individuals to associate and bond with similar others), and the types of tie formations and decays [9-11]. These frameworks can be applied to characterize medical communities. In a recent study, SNA was applied in an investigation of the network characteristics of the group Health Care Social Media Canada (HCSMCA), particularly as they relate to the formation of an online community [12]. Beyond community formation, an assessment of the network structure can help characterize the actual and potential flow of information between different professional physician groups. The characterization of social activity and information flow is a first step toward understanding the visibility of each network within the online medical community and the potential to transmit health information rapidly and effectively.

Twitter can be thought of as an information sharing network because of its highly skewed distribution of followers, or listeners, and its low rate of reciprocated connections (most information sharers are not followers of their followers) [11]. A number of computer science studies have attempted to characterize how far and how quickly information flows on Twitter [11-13]. This general idea is referred to as information diffusion. It is important to first describe a few models of information diffusion to better understand information diffusion in the context of online medical communities.

### Connectivity Variation Across Network Models

#### Overview

**Figure 1:**
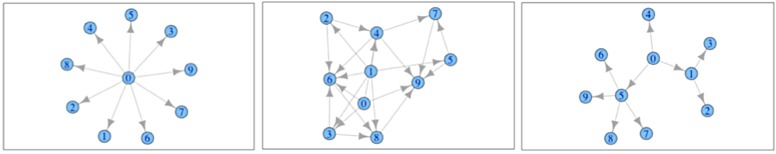
Network configurations - Star, Random, Small World (left to right).

There are several network topologies (structures or models of the network) that highlight common information diffusion patterns. Figure 1 shows examples of three of these topologies for small directed networks. In each example, the circles are referred to as “nodes” and the lines as “edges”.

The number of edges connected to a node is referred to as the degree of the node. The direction of the edge in each network indicates the direction of information flow. The difference between these networks is the degree distribution, ie, the number of incoming and outgoing edges of each node in the network. The pattern of these edge connections defines the structure of the network and dictates how quickly a message travels.

#### Star Networks

In star networks, the degree distribution of the nodes is heavily skewed. Every time node 0 sends a message, every node in the network receives it immediately. However, when node 8 sends a message, no one ever receives it. This is equivalent to a Twitter subnetwork where a Twitter account or Twitter user has followers who do not connect to each other (they do not have a direct communication channel to each other). When users in a subnetwork are well connected, we say that they form a “cohesive community”. The amount of cohesion is defined as the number of common neighbors a group of individuals have, divided by the total number of neighbors. This measure is a variant of a more traditional local “clustering coefficient” measure. A clustering coefficient is user specific and measures the number of triangles a user is involved in. Because connectivity is limited in a star network (no neighbors have edges between them), it does not form a cohesive community. However, for messages sent from the center node, this network is optimal for basic information dissemination since everyone receives the message right away and no extra messages are sent. Messages and ideas sent from the periphery though, do not spread. At the same time, while star structures within a real world network are ideal for quick information diffusion from the center node, they are problematic in terms of network resilience and community development.

#### Random Networks

In random networks, the degree of the nodes follows a normal distribution. It is unusual to see a node that has a very high or low degree and the dissemination power of each node tends toward the mean. When node 0 sends a message, it takes three steps, or three hops, before it reaches everyone in the network. When node 1 sends a message, it reaches everyone in two hops. If everyone who receives the message sends it forward, some nodes will receive the message multiple times. Since extra messages are being sent, the dissemination is not considered efficient. In other words, because of the random connectivity pattern, spreading a message requires more individuals to participate and is thus not efficient. Further, this network is not a cohesive community since only a small number of neighbors are connected to each other. In general, information networks, social networks, epidemics, and other such networks exhibit non-random connectivity patterns [14].

#### Small World Networks

In small world networks, the diameter of the graph (the furthest distance between any two nodes in the graph) is low and the amount of cohesion is higher than in a random network. A network in which the degree of the nodes follows a power law distribution indicates a small world network. In this network, a few nodes are very well connected, but most are not. In our example, if node 0 sends a message, it takes two hops to reach everyone without extra messages (in a larger example, we would expect a small number of redundant messages). This network is more efficient than a random network. Well-connected users in this network have comparatively high dissemination power and act as hubs, but messages from the periphery can also be efficiently disseminated because the diameter of small world networks is low. This means that even though most nodes are not neighbors of each other, the number of hops needed to reach every node is small. Small world networks tend to have pockets of cohesive communities throughout the network. Another interesting property of small world networks is that they are more resilient to removal of random nodes from the network than are random networks. Because most random nodes will have a small degree, deleting them will not increase the diameter or decrease the cohesion/clustering coefficient significantly [15].

Many networks have been shown to follow small world properties, including social networks, protein networks, and voter networks. When celebrities are excluded, the degree distribution of nodes on Twitter approximates a power law distribution [14].

#### Potential Information Dissemination Based on Network Structure

A network with a power law structure has the theoretical capacity to spread information, even arising from the periphery, efficiently to many users. In generated networks of this type, a message can be disseminated to everyone in the network using a simple dissemination strategy and a small number of resends (logarithmic in the network size) [16]. Turning to the specific dynamics of Twitter in which followers observe messages from those they follow and then decide whether or not to retweet them, a common method to capture this behavior is the independent cascade model [17]. In this model, each person resends a message with some fixed independent probability, which captures the likelihood they will find a message interesting. In this setting, for generated power law graphs (which have structures similar to Twitter), once these probabilities pass a certain reasonable threshold, there is a high probability that a message dissemination will become a “long-lived” epidemic [18]. Researchers [19] provided a technique for identifying influential individuals in this model, ie, users who, due to their position in the network, are likely to instigate large information cascades. In their simulations on a real social network topology of a collaboration network among physics researchers, they showed that if a message was resent 10% of the time, termed a “uniform resend probability” of 10%, they could identify a message source that would cause a message to spread to thousands of other users. Kwak et al [13] analyzed message spread on a 2009 snapshot of Twitter containing 41.7 millions user profiles and 106 million tweets and found that over 96% of tweets were not retweeted and that the tweets with the highest dissemination during this period were generally retweeted by 12% to 30% of the sender’s followers. However, even with fewer than 1000 followers, if a message began disseminating quickly, information cascades were much larger than the size of the original follower’s network. This was the case even when the number of initial retweeters was small.

To summarize, computer science theory on information dissemination elicits two things about networks with the properties observed of the Twitter follower graph: (1) Twitter resembles a small world graph with a degree distribution that follows a power law distribution, (2) dissemination to a large number of nodes in a small amount of time is possible, and (3) these large scale disseminations can be achieved with simple resend rules (ie, they do not require sophisticated centralized planning).

It should be mentioned that there is a natural trade-off between information dissemination and community cohesion. If there is high community cohesion, members of the community will have quick access to information. However, members outside of the community will not. In contrast, if cohesion is low, information can disseminate to a broader audience. However, the actual amount of dissemination in a subnetwork without active community participants can be low. For information transmission networks, developing a community with moderate cohesion will increase the dissemination of information to a broader audience.

In this preliminary study, we sought to employ applied graph theory and a basic SNA framework in order to characterize and understand information diffusion on social media within a subset of the medical community by examining the Twitter networks of a few medical professional societies.

## Methods

### Overview

Social network analysis and network configuration models were used to characterize community structure and information dissemination of four professional physician groups that have a presence on Twitter. The core groups in this analysis are: the American Medical Association (AMA), the American Academy of Family Physicians (AAFP), the American Academy of Pediatrics (AAP), and the American College of Physicians (ACP). Explanations of the metrics used in this study are presented in Table 1.

**Table 1:** Description of metrics from Twitter.

Metric	What it measures	Description and purpose of metric
Number of followers	Actual information dissemination	How many people/groups received your message? AND How many people/groups may resend (retweet) your tweet? A larger number indicates that a higher probability exists for retweeting the message. Therefore, the potential for large-scale information flow increases.
Number of Level 2 followers	Level 2 information dissemination potential	How many followers (active listeners) do your followers have? This number represents the number of Level 2 followers who will see your tweet if all of your followers retweet it and none of your followers have the same Level 2 followers.
Dissemination network size	Information dissemination potential	How many people can see your message if all of your followers retweet it? This number represents the followers and the Level 2 followers who see your tweet if all of your followers retweet the message and none of your followers have the same Level 2 followers.
Number of information sharers	Active sources of information	Who are the other people or groups on Twitter that you are getting information from? A larger number indicates more information sources to retweet messages from. These are active sources because they send you information.
Number of tweets	Frequency of information disseminated	How often do you share information with your followers? A large number indicates that you regularly post tweets/re-tweets for your followers to view.
Number of retweeters	Actual number of information disseminators	How many people retweeted a particular tweet you sent? A large number indicates that many people shared your tweet with their followers.
Retweeter network size	Number of Level 2 followers	How many people receive the tweet when some of the followers retweet it?

### Network Characterization

The Twitter API (api.twitter.com) was used to characterize the network: determine the number of followers and the number of tweets for each of these groups. These data were collected between July 2012 and September 2012. Accounts that were disabled, private, or not recognizable by our automated programs were ignored. This amounted to less than 1% (1257/238,853) of the accounts we had access to. Similar to other studies in computer science, the number of followers was used as an indicator of actual information dissemination, the Level 2 followers as an indicator of the information dissemination potential, the number a user is following as a way to identify potential sources of information, and the number of tweets as an indicator of the frequency of information dissemination for a particular group or individual.

We approximated community cohesion for this dataset by measuring the amount of overlap in followers between the professional groups as a percentage [(A intersect B)/min(|A|,|B|)*100]. Overlap is necessary to develop a cohesive community that has common sets of followers and connections between subsets of the followers. As previously mentioned, too much overlap may reduce the amount of information that disseminates outside of the community. We also used visualization to better understand this overlap.

### Information Flow

The actual information flow of the tweets for each of these groups for a one-month period from August 1, 2012 to September 1, 2012 was analyzed. Tweets sent by the four professional physician groups were examined using Topsy, a Twitter data aggregator [20], to determine how many times each tweet was retweeted. Topsy is a Twitter partner that has indexed all public tweets since Twitter was founded in 2006. Of note, since this data is dynamic, more retweets can occur after the data has been collected.

### Tweet Propagation Analysis

For each group, tweets sent between July 1, 2012 and September 12, 2012 were identified and assessed, including the number of retweets and details of their dissemination. This was followed by the identification of individuals who retweeted the message, the determination of the number of followers for each retweeter and the computation of the retweeter network size to measure actual information flow. Looking at how many times a message was retweeted, compared to the number of times it could have been retweeted if all of an organization’s followers retweeted the message, provides a measurement of how close the actual tweet dissemination is compared to the theoretical best.

## Results

### Network Characterization

Table 2 shows the number of followers, the number a user is following, the number of tweets, and the information dissemination potential for each professional medical group in this analysis. These statistics show that all of these professional groups have thousands of followers and a theoretical information dissemination potential ranging from 6.9 to 122 million people. This value is the sum of each professional group’s followers and Level 2 followers. For example, if all the followers of the AAFP retweet a message sent by the AAFP, 6,959,092 people will see the message. Given the large potential for dissemination, we can view each of these professional groups as information brokers within the Twittersphere.

**Figure 2:**
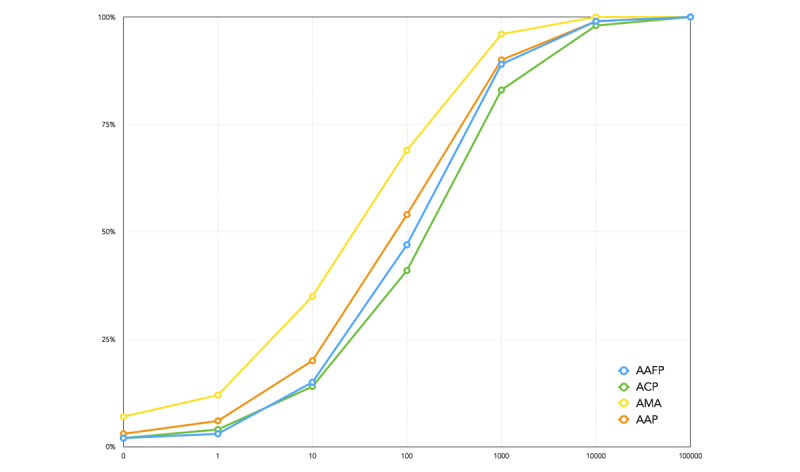
Information dissemination potential for each professional physicians group - American Medical Association (AMA), American Academy of Family Physicians (AAFP), American Academy of Pediatrics (AAP), and American College of Physicians (ACP).

The information dissemination potential for the followers of each group was plotted using a cumulative frequency graph in Figure 2. Each line represents one organization and shows cumulatively what percentage of their followers has a given size follower network. Of note, the graph is a log-scale. For example, over 25% of AMA followers have fewer than 10 individuals following them. We see that the distribution of followers for these subnetworks is consistent with a power law distribution, with approximately half the nodes having fewer than 100 followers and very few nodes having hundreds of thousands of followers.

In AAFP and ACP, it emerged that over half of the followers have a strong listener network (Level 2 follower network) with at least 100 listeners. The median number of listeners for each of the followers of AAFP, ACP, and AAP are 120, 165, and 81 respectively. In contrast, the majority of followers of AMA have smaller (quieter) listener networks, with over 50% (119,560/213,122) having fewer than 50 listeners. The potential to disseminate widely exists for each of these organizations since all of the organizations have a large percentage of listeners who are themselves information brokers.

**Figure 3:**
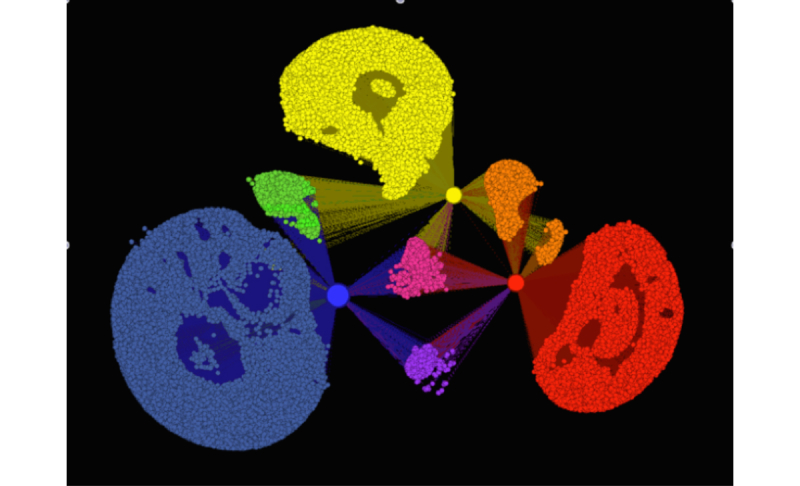
Follower network of American Academy of Family Physicians (yellow), American College of Physicians (red), and American Academy of Pediatrics (blue). Size of group nodes based on number of followers.

A detailed social network visualization of the networks’ overlap is shown in Figure 3. This visualization only shows the three smaller physicians group networks since the largest one, the AMA, has approximately ten times the number of followers as the other three networks combined.

In the networks under study, there is evidence of the beginnings of a collective community, where large percentages of individuals (13%-55%) are following more than one of the professional groups. As the illustration shows, the majority of followers are specific to one of the groups. The pink nodes are following all three of the professional groups, while the green, orange, and purple nodes are following two of the professional groups. The overall common overlap across all four groups is only 471 individuals, a very small percentage of the overall networks for these groups, indicating a limited amount of community cohesion and cross-fertilization, but still allowing for efficient channels (fewer redundant messages) for information dissemination.

**Table 2:** Dissemination potential and professional group statistics during the study period.

Professional group	Number of followers	Number following	Number of tweets	Information dissemination potential
American Academy of Family Practice (AAFP) (@AAFP)	7546	298	2788	6,959,092
American College Physicians (ACP) (@ACPinternists)	5955	2023	2979	11,228,160
American Academy of Pediatrics (AAP) (@AmerAcadPed)	11,768	132	1184	14,496,559
American Medical Association (AMA) (@AmerMedicalAssn)	213,122	5729	7065	122,066,397

### Information Flow

**Figure 4:**
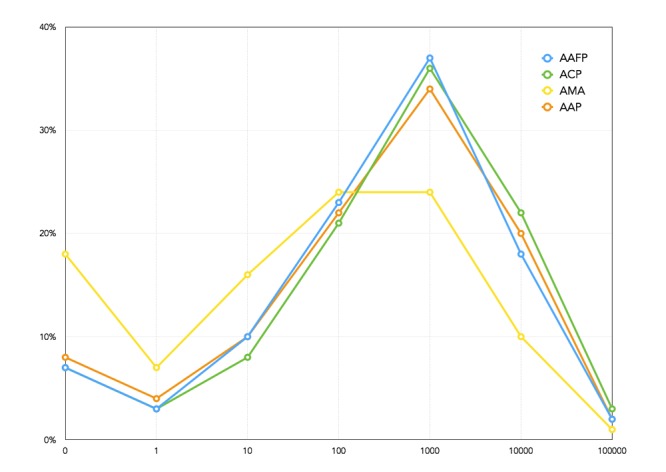
Number of tweets/retweets sent by followers of the four professional groups - American Medical Association (AMA), American Academy of Family Physicians (AAFP), American Academy of Pediatrics (AAP), and American College of Physicians (ACP).

When considering information diffusion potential, it is reasonable to exclude followers who have never tweeted or retweeted, since it is likely that those individuals will not retweet a message from one of the professional groups. The more tweets a group’s followers send, the higher the likelihood for larger dissemination. Figure 4 shows a continuous histogram of the volume of tweets of followers for each of our professional groups. The x-axis shows logarithmically the volume of tweets sent by followers of each professional group. The y-axis shows the percentage of followers who have sent that volume of tweets. For example, the percentage of followers that have not sent any tweets is 6.92% for AAFP (522/7546), 8.17% (962/11,768) for AAP, 7.22% (430/5955) for ACP, and 18.43% (39,275/213,122) for AMA.

If these followers and their Level 2 followers are removed from the dissemination network, the overall information dissemination potential decreases by less than 1% for all four professional groups. This indicates that the followers who do not send any tweets/retweets have a small number of followers themselves and are not essential information brokers. When all the followers who have sent only 10 or fewer tweets are removed, then the AMA professional group information dissemination potential is reduced by over 35%. The other professional groups are still impacted by less than 1%. This is an indication that the AMA followers’ network is not as active as the other three professional networks. For the other three groups, there is a stronger correlation between the number of tweets disseminated and the number of followers.

### Tweet Propagation Analysis

**Figure 5:**
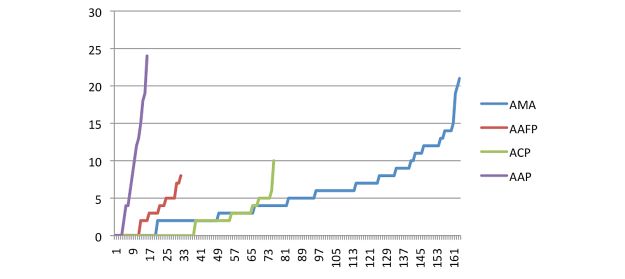
Number of retweets for messages sent in August 2012. [American Medical Association (AMA), American Academy of Family Physicians (AAFP), American Academy of Pediatrics (AAP), and American College of Physicians (ACP)].

In addition to the information dissemination potential, the actual retweet propagation of a sample of tweets was assessed. Figure 5 shows the propagation of actual tweets during the month of August 2012 (ie, the number of retweets for each message sent by the different professional medical associations.)

The x-axis represents each tweet where the tweets are sorted by number of retweets. The y-axis represents the number of retweets. The AMA posted the largest number of tweets (164), while the AAP posted the fewest during this time period. Each organization had a number of tweets that were not retweeted by anyone. The largest number of retweets for any of these organizations during this month was 24. Given that each of these groups has thousands of followers, this level of retweeting leads to information dissemination that is far below the information dissemination potential shown in Table 2. While unlikely, even with a small number of followers retweeting a message, the diffusion can still be large at the third and fourth hop. We have not computed the third and fourth hop networks here, but previous literature supports this pattern of diffusion; Bakshy et al [21] analyzed a data set from 2009 containing 1.6 million Twitter users in 2009 and identified common information cascade patterns of their tweets. Many of these patterns involved transmission with third and fourth hop users. Therefore, we cannot discount that dissemination occurs beyond the Level 2 followers.

Finally, the dissemination of a particular tweet was considered: how does the dissemination of an actual tweet compare to the theoretic best? Here, we focus on the propagation of the tweet as opposed to the content of the tweet. Are there any tweets that are disseminating to a large fraction of this medical community? Table 3 compares the tweet with the highest retweet dissemination (actual information dissemination) for tweets sent between July 1, 2012 and September 12, 2012 to the dissemination potential for each professional group.

Overall, the number of retweets and the number of individuals who received the tweet is less than 0.2% of the total population dissemination potential, with the tweet from the ACP disseminating the least.

**Table 3:** Top tweets for each professional group.

Professional group	Tweet	Number of retweets	Actual information dissemination	Fraction of information dissemination potential
AAFP	“Ask your Doctor if medical advice from a TV commercial is right for you…”	10	9558	0.00137
ACP	“Interaction between proton-pump inhibitors clopidogrel clinically unimportant…”	7	489	0.000044
AAP	“Tragedy in CO – in the wake of news about another act of gun violence, how to talk with children and teens…”	25	25,482	0.00176
AMA	“September is Women in Medicine Month, a time to celebrate growing number, influence of women physicians	45	200,778	0.00164

## Discussion

### Principal Findings

At the time of our study, the AMA had the largest number of followers—and thus, information diffusion potential—and was trailed by the AAP, AAFP, and ACP, respectively. However, each of the smaller organizations had a strong network of followers among which were individuals who themselves are potentially strong information brokers. We also began to see interconnectedness among these groups as evidenced by a group of users who follow all three smaller organizations. This preliminary analysis shows possibly large information diffusion potential, yet when we analyzed actual tweets sent, the actual dissemination was well below the calculated potential.

With the growing popularity of social media and Twitter, medical organizations are urged to engage in social media and actively share information. Therefore, it is important to determine what metrics can be used to measure the effectiveness of this as a medium. This study attempted to describe the characteristics of four medical networks and analyze their theoretical information dissemination potential, their actual information dissemination, their information sharers, and their propagation and distribution of tweets.

### Limitations

This study has several weaknesses. First, we captured our data at one point in time. As such, it is only a snapshot of the Twitter networks described. Social media networks tend to be dynamic with followers added and dropped from moment to moment. So, in all likelihood, these networks may look different today than they did during the study period. Additionally, the overall trend on Twitter is expansion, with increased number of users. In fact, at the time of manuscript revision submission, each of the organizations described in this manuscript has shown to have a much larger following. This also applies to the data captured for the individual tweets, which may have diffused beyond the study period. Second, our analysis does not allow for an investigation of the inter-activity, interactions, and engagement within the networks. As a result, it is impossible to draw conclusions about motivation for dissemination or how content drives diffusion. Third, though the total percentage of accounts we ignored in our analysis was less than 1%, we could not determine the percentage of private accounts that we did not have access to. According to Beevolve [22], in 2012, approximately 12% of Twitter users had private or protected accounts. Finally and most importantly, the dissemination potential described should be interpreted with caution. The number of followers may not necessarily mean each follower is a valid or relevant one, or one that can further propagate the message. Many followers may be family members, friends, commercial entities, organizations, or individuals with no interest in the topic. Further studies will need to look at the specific nature and identity of the followers. Additionally, because information is disseminated on Twitter in a continuous manner, there is no guarantee that all the followers will receive or will have seen all the messages, as patterns of use vary and many followers check their accounts in a more sporadic manner, thus “missing” many tweets in real time (and, as a result, the opportunity to retweet or act on the message in other ways).

This work is merely the first step toward understanding the information power and potential of several medical professional groups on Twitter.

### Conclusions

This analysis indicates that these medical groups participate in subnetworks with small world type tendencies. This structure allows for large-scale information dissemination; however, actual dissemination is well below potential for all four professional groups. This is consistent with many other groups on Twitter. Large-scale information diffusion in Twitter is driven by information brokers who have at least a moderate number of followers, some of whom are active followers. In other words, it is more valuable to a network to be well connected to a few influential information brokers than to have a large number of first degree followers. Although having a large number of followers is beneficial, small networks can still achieve a high potential distribution if they have a few information brokers who themselves are active and well connected. Developing a community that is active (in terms of retweeting) and engaged (in terms of content, mutuality, and reciprocity) is important for strong dissemination. As demonstrated in a previous study [23], social media engagement is a complex system to describe and measure. Engagement may be thought of as a continuum (low-medium-high) and may follow specific patterns and hierarchical structures. Indeed, one metric of engagement, as described in Neiger et al, is retweeting. While these professional groups all have some individuals who retweet heavily, those individuals were not very engaged during our study period. Encouraging these information brokers to retweet the shared content will help increase dissemination. Other ways to increase dissemination include tweeting more regularly (increasing the volume of tweets to show active engagement in the Twitter platform) and retweeting more often. Others will be less interested in retweeting content if the organizations themselves do not retweet content. These strategies will help develop a more cohesive community of shared followers, for cross-fertilization of information.

The content of the messages is of course of utmost importance. Even with strong channels for dissemination, tweets must be timely and engaging in order to provide the hook for followers to retweet and begin to reach the vast potential audience.

In the past few years, reports have been published about the use of Twitter for various purposes within the field of medicine: as a support tool for patients with chronic conditions [24], by ministries of health for health promotion [25], as an educational tool during conferences [26], to track public health message diffusion [27], and as a teaching tool in health profession education [28-34]. A few researchers are developing metrics that may enable this medium to assist in measuring journal article impact [35] and to predict citations of journal articles [36]. However, as a field of research, Twitter usage—the flow of information, content analysis, profiles of message generators and of message recipients, overall effects on public behavior—is still in its infancy.

This study is one example of the development of theoretical models of knowledge dissemination that could have practical implications in how we use this medium to empower patients, disseminate important public health messages, or promote our ideas and specialties. As researchers attempt to characterize best practices in the use of social networks for knowledge transfer and dissemination, they will need to look at the networks themselves, conduct content analysis of the messages, and assess the behavior and actions of the messengers as well as the recipients. This calls for more multi-disciplinary research, involving experts in computer science, communications, linguistics, and cultural studies, to develop and advance this field of inquiry.

## Abbreviations

AAFP: American Academy of Family Physicians
AAP: American Academy of Pediatrics
ACP: American College of Physicians
AMA: American Medical Association
SNA: social network analysis
